# Plasma proteomic and autoantibody profiles reveal the proteomic characteristics involved in longevity families in Bama, China

**DOI:** 10.1186/s12014-019-9242-4

**Published:** 2019-05-20

**Authors:** Shengliang Ye, Li Ma, Rong Zhang, Fengjuan Liu, Peng Jiang, Jun Xu, Haijun Cao, Xi Du, Fangzhao Lin, Lu Cheng, Xuefeng Zhou, Zhihui Shi, Yeheng Liu, Yaojin Huang, Zongkui Wang, Changqing Li

**Affiliations:** 1 Institute of Blood Transfusion, Chinese Academy of Medical Sciences and Peking Union Medical College, Chengdu, 610052 China; 2 Shanghai RAAS Blood Products Co. Ltd, Shanghai, 201401 China; 3 Bama Plasmapheresis Center, Hechi, 547500 China

**Keywords:** Bama longevity area, Plasma proteomics, Autoantibody profiles, Tandem mass tag, Human proteome microarray

## Abstract

**Background:**

Chinese Bama Yao Autonomous County is a well-known longevity region in the world. In the past 30 years, population and genome studies were undertaken to investigate the secret of longevity and showed that longevity is the result of a combination of multiple factors, such as genetic, environmental and other causes. In this study, characteristics of the blood plasma proteomic and autoantibody profiles of people from Bama longevity family were investigated.

**Methods:**

Sixty-six plasma donors from Chinese Bama longevity area were recruited in this study. Thirty-three offsprings of longevous families were selected as case studies (Longevous group) and 33 ABO (blood type), age, and gender-matched subjects from non-longevous families were selected as controls (Normal group). Each group contains 3 biological replicates. Tandem mass tag-based proteomic technique was used to investigate the differentially expressed plasma proteins between the two groups. The auto-reactive IgG antibody profiles of the 3 pooled samples in each group were revealed by human proteome microarrays with 17,000 recombinant human proteins.

**Results:**

Firstly, 525 plasma proteins were quantified and 12 proteins were discovered differentially expressed between the two groups. Secondly, more than 500 proteins were recognized by plasma antibodies, 14 proteins ware differentially reacted with the autoantibodies in the two groups. Bioinformatics analysis showed some of the differential proteins and targeted autoantigens were involved in cancer, cardiovascular disease and immunity.

**Conclusions:**

Proteomic and autoantibody profiles varied between the offspring of longevous and normal families which are from the same area and shared the same environmental factors. The identified differences were reported to be involved in several physiological and pathological pathways. The identified proteins will contribute to a better understanding of the proteomic characteristics of people from Bama longevous area and a revelation of the molecular mechanisms of longevity.

**Electronic supplementary material:**

The online version of this article (10.1186/s12014-019-9242-4) contains supplementary material, which is available to authorized users.

## Background

Human longevity is a complex phenotype with a significant familial component [1, 2]. Genome studies were taken and indicates that gene polymorphisms contribute to human longevity in the past years [3–5]. However, only examining the genome profiles does not give an integrated view, as proteome may not be totally accurately predicted by gene profiles due to several factors [6]. Furthermore, proteins are major components of the tissues and cells with diverse regulatory, enzymatic and structural functions [7, 8]. In the last 20 years, proteomics analysis has been widely used to find biomarkers, proteins that change in concentration or state in associations with a specific biological process or disease [9–12]. Blood plasma, which include thousands of proteins, is a predominant sample used for human proteomics study.

Bama Yao Autonomous County, a remote and mountainous county located in the northwest of Guangxi Zhuang Autonomous Region (Guangxi province) of China, is known for its longevity [13, 14]. The longevity township standard set by the United Nation is 7 centenarians among 100,000 people [13]. The population size and centenarian rate in Bama County are around 240,000 and 30/100,000 respectively, according to the National Population Census of China in the past decades [14, 15]. In 2003, Bama was awarded the certificate of The World’s Fifth Longevity Township by the International Natural Medical Association [13]. In the past decades, the people of Bama have been utilized for aging/longevity cohort [16–19].

To investigate the secret of Bama-induced longevity at the proteome levels, in this study, 66 plasma donors from Bama longevity hotspot which shared the same environmental factors were recruited and divided into two groups, offsprings of longevous families and offsprings of non-longevous families. Tandem mass tag (TMT)-based approach was used to systematically investigate the expression of proteins in the plasma of the two groups. The differentially expressed proteins (DEPs) were revealed by bioinformatics analysis.

In most proteomic profile studies, the high abundance proteins in plasma (include albumin, immunoglobulin G, transferrin, etc.) were removed to greatly improve detection of lower abundance protein [10, 20–23]. However, IgG antibodies are an important part of the immune system, which plays a vital role in physiological conditions in healthy individuals. Autoantibody or self-reactive antibody refers to antibody that are present in the blood plasma of individuals in the absence of deliberate immunization with the target antigen, it reacts with human own proteins or tissues [24]. It is generally believed that autoantibodies are critical for many physiological and pathological processes [25, 26]. In this study, the HuProt arrays, comprised of ~ 17,000 individually purified human proteins, were also employed to investigate the IgG autoantibody repertoires of the two groups of people from Bama.

## Methods

### Sample preparation

A questionnaire was sent to healthy plasma donors in the Bama longevity area, and the number of long-lived immediate family members (≥ 85 years old) within three generations in the donors were investigated. A donor with at least three long-lived immediate family members was defined as the offspring of longevous family, and donors without an immediate family member ≥ 85 years old within three generations were offsprings of non-longevity families.

In total, 33 offsprings of longevous families were recruited as case studies (Longevous group). And also 33 ABO-, age- and gender-matched offsprings of non-longevity families were recruited as controls (Normal group). The inclusion criteria of the participators were recruited as previously described and apheresis plasma of the participators was used in this study [27]. Briefly, all participators were over 18 years of age, healthy and unrelated. People who had prior history of thrombus or hemorrhage, usage of oral anticoagulation therapy, hepatic disease, HIV infection, pregnancy, diabetes, cardiopathy, hypertension, renal insufficiency, respiratory diseases and others were excluded from this study. Pooled plasma samples were generated by combining equal volumes of each 11 individual plasma samples from each group. The basic information of the volunteers was shown in Table 1. This study was approved by the Ethics Committee of the Institute of Blood Transfusion (No. 201716). In accordance with the Declaration of Helsinki, all participants gave informed consent prior to their entering the study.

**Table 1:** The basic information of the study cohort

Cohort no.	Longevous (offsprings of longevous families, n = 33)			Normal (offsprings of non-longevity families, n = 33)		
	A1	A2	A3	B1	B2	B3
	(n = 11)	(n = 11)	(n = 11)	(n = 11)	(n = 11)	(n = 11)
Mean age	45.45	45.73	46.64	46.09	46.45	46.09
(Age range)	(34–55)	(35–56)	(36–56)	(33–56)	(37–55)	(35–54)
Gender						
Female	6	7	7	6	7	7
Male	5	4	4	5	4	4
Blood type						
A	3	3	3	4	3	3
B	4	2	3	1	3	4
AB	0	1	0	1	0	1
O	4	5	5	5	5	3

### TMT-based quantitative proteomic analysis

#### Tandem mass tagging labeling and HPLC fractionation

The high abundance proteins that account for approximately 80% of the total plasma protein concentration were removed from the pooled plasma samples, using a ProteoMiner™ Protein Enrichment Introductory Large-Capacity Kit (Bio-Rad, Richmond, USA) according to the manufacturer’s instructions. After being reduced with 5 mM DL-dithiothreitol for 30 min at 56 °C and alkylated with 11 mM iodoacetamide for 15 min in darkness at room temperature, the protein sample were digested with trypsin at a trypsin/protein mass ratio of 1:50 overnight in the first stage and 1:100 for 4 h in the second stage. Approximately 100 µg protein for each sample was digested with trypsin for the following experiments. Tryptic peptides were desalted by Strata X C18 SPE column (Phenomenex, CA, USA) and labeled with a 6-plex TMT kit (Thermo Fisher Scientific, CA, USA) according to manufacturer’s instructions. The 6 labeled samples then fractionated by high pH reverse-phase HPLC using 300Extend C18 column (5 µm particle size, 4.6 mm ID, 250 mm length, Agilent, CA, USA). Peptides were eluted with a gradient of acetonitrile (8% to 32%) in 10 mM ammonium bicarbonate (pH 9.0) over 60 min. At last, 60 fractions were collected, and they were combined into 18 fractions and dried by vacuum centrifuging.

#### LC–MS/MS and bioinformatics analysis

Samples were measured using LC–MS instrumentation consisting of an EASYnLC 1000 ultra-high-pressure system coupled via a nano-electrospray ion source to a Q Exactive™ Plus (Thermo Fisher Scientific). Purified peptides were separated on a reversed-phase analytical column (150 mm length, 75 μm ID). For each LC–MS/MS analysis, the electrospray voltage applied was 2.0 kV. Intact peptides were detected in the orbitrap at a resolution of 70,000, and ion fragments were detected in the orbitrap at a resolution of 17,500. In the MS survey scan, a data-dependent mode with an automatic alteration (1 MS scan followed by 20 MS/MS scans) was used for the top 20 precursor ions above a threshold ion count of 5 × 10^4^ with 30 s dynamic exclusion. Automatic gain control was used to prevent overfilling of the orbitrap; 5 × 10^4^ ions were accumulated for generation of MS/MS spectra. For MS scans, the m/z scan range was 350 to 1800, and the fixed first mass was set as 100 m/z.

MS/MS data was analyzed by MaxQuant software with integrated Andromeda search engine (Version 1.5.2.8). The mass tolerance for precursor ions was set as 20 ppm in First search and 5 ppm in Main search, and that for fragment ions was set as 0.02 Da. False discovery rate (FDR) was adjusted to < 1% and minimum score for peptides was set > 40. Only unique peptides were used for protein quantification. And for protein quantification method, TMT 6-plex was selected in Mascot. FDR was adjusted to < 1% at protein, peptide and peptide spectrum match (PSM) level. Gene Ontology (GO) annotation (the biological process, cellular component and molecular function) of the differentially expressed proteins were derived from the UniProt-GOA database (http://www.ebi.ac.uk/GOA/). PSORT/PSORT II and SubLoc (http://www.bioinfo.tsinghua.edu.cn/SubLoc/) were used to predict subcellular localization of all identified DEPs. A *p* value < 0.05 was used as the threshold to determine the significant enrichments of GO annotation.

### Plasma autoantibody profiling using the human proteome microarrays

#### Human proteome microarrays

The HuProt™ V3.1 microarray (CDI Laboratories, Inc. Mayaguez, USA) composed of about 17,000 recombinant human proteins was used in this study. All of the recombinant human proteins were generated by the *Saccharomyces cerevisiae* expression system and carried an N-terminal glutathione S-transferase (GST) tag. Each protein was spotted in duplicate [11, 28]. The microarray was firstly blocked with blocking buffer (5% BSA in 0.1 mol/l TBS-T, pH = 7.5) at room temperature for 1.5 h. Then it was washed with with TBS-T and then Milli-Q water for 10 min respectively. The six pooled plasma samples of two groups were diluted 1: 2000 in 0.01 mol/l Tris-Buffered saline containing 0.1% (v/v) Tween 20 detergent (TBS-T, pH = 7.5) with 5% BSA. Five ml of diluted plasma were added and incubated with microarray under a glass coverslip at room temperature for 1 h. After rinsing with TBS-T and then Milli-Q water, Cy3-conjugated anti-human IgG diluted 1:2000 in TBS-T was added to the washed microarray and incubated in darkness at room temperature for 1 h. The microarray was then washed again as described above. After drying by centrifugation (ChipMate PMC-082, Tomy, Japan), microarray was subjected for scanning with the Axon GenePix 4000B microarray scanner (Molecular Devices, CA, USA) using an excitation wavelength of 523 nm and an emission filter specifically for cy3 fluorescent dye. The optical signal was detected by a photomultipliers tube (PMT).

#### Human proteome microarray data analysis

Human proteome microarray data analysis GenePix Pro 6.0 software (Molecular Devices, CA, USA) was used to obtain microarray signal intensity. The foreground median intensity (F median) and background median intensity (B median) for each spot on the microarray images were acquired. To quantify the signal intensity for each spot, we calculated the signal intensity for each spot, which was defined as the foreground median intensity divided by its local background median intensity (F median/B median). The intra-array signal intensity normalization and positive hits identification were taken as described in the previous reports [29, 30]. Briefly, the signal intensities of the spots within a protein microarray were normalized by setting the median intensity of that microarray equal to 1. To identify autoantibodies that bind to a protein on microarray (positive hits), an intensity cutoff value needed to be assigned for each microarray. We used I to denote the normalized intensity of a spot (control spots omitted) on the protein microarray. And Mean_i_ denote the average of I (control spots omitted) on a protein microarray. The standard deviation (SD) away from the mean of the signal intensities for all the spots in a microarray was calculated. A cutoff was defined as Mean_i_ + 6SD, and spots producing a signal intensity greater than the cutoff were identified as “positive hits.” Moreover, each protein was printed in duplicate on a microarray, proteins and controls were considered as a positive feature only when both of their duplicate spots were simultaneously judged as a positive hit. T-test was chosen to assess the differential significance for each protein between the two groups based on signal value. The Database for Annotation, Visualization and Integrated Discovery (DAVID) v6.8 were used for the functional category analyzation of the identified autoantigens, subcellular localization prediction and GO annotation of the different autoantigens between groups.

## Results

### Differentially expressed proteins revealed by quantitative proteomic analysis

**Fig. 1:**
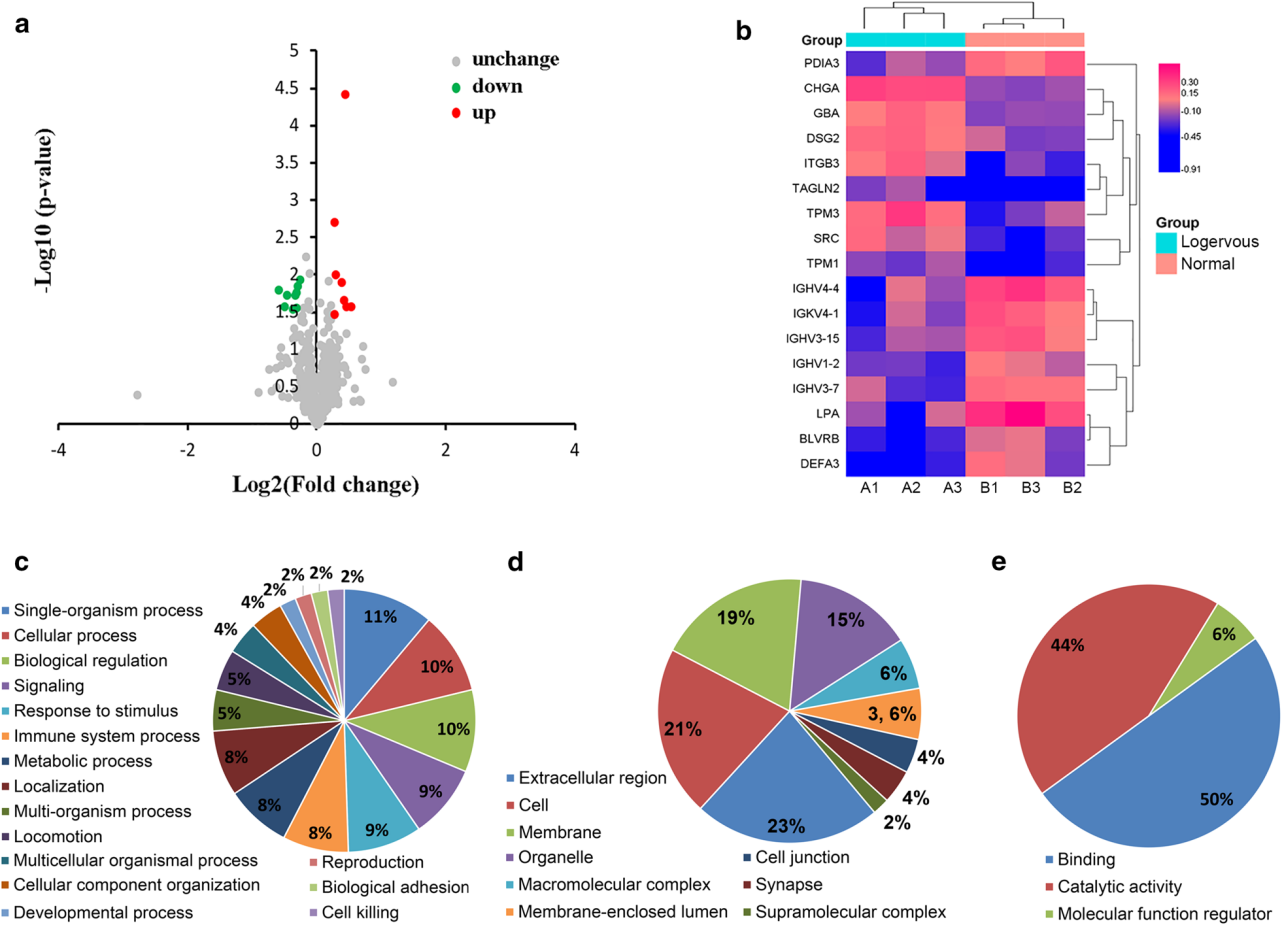
Differentially expressed proteins in the longevous and normal groups. TMT-based proteomic technique was used to study the protein characteristics in longevity families. Differentially expressed proteins and were defined as having fold changes > 1.2 (> 1.20 increased or < 0.83 decreased). A two tail Student’s T-test was performed and a p-value < 0.05 was considered to be statistically significant**. a** The volcano plot shows the up- (red) or down-regulated (green) proteins between the two groups. **b** Hierarchical clustering of differentially expressed proteins. The color scale bar locates in the bottom, and green and red indicate decreased and increased levels of the identified poteins, respectively. A1, A2 and A3, 3 replicates of the longevous group; B1, B2 and B3, 3 replicates of the normal group. **c** Biological processes, **d** cellular components and **e** molecular functions of GO annotation based function classification of the differentially expressed proteins

A total of 598 proteins were identified using MaxQuant, among which 525 proteins were quantified at more than a 95% CI, and proteins with no less than two unique peptides were considered to be a positive identification (see Additional file 1). For comparison between the longevous and normal groups, a protein featuring a fold change of > 1.2 or < 0.83 and a p value of < 0.05 was regarded as DEP. The identified DEPs were showed by a volcano plot and a hierarchical clustering heat map (Fig. 1a, b), and described in Table 2. 17 DEPs were identified based on the criteria, which were corresponding to 12 different proteins, as some results corresponded to different type or different isoforms of the same protein (DEPs No. 3–7, No. 10 and 11). Among these 12 proteins, 5 were significantly down-regulated (LPA, DEFA3, immunoglobulin, BLVRB and PDIA3), 7 were accumulated (TPM, GBA, DSG2, SRC, CHGA, ITGB3 and TAGLN2) in the longevous group A than normal group.

**Table 2:** Differentially expressed proteins in the longevous and normal groups detected by TMT-based proteomic technique

No.	Name	Ratio^a^	P-value^a^	Protein description	MW^b^ (kDa)
1	LPA	0.66	0.016	Lipoprotein A	501.31
2	DEFA3	0.708	0.026	Defensin 3	10.245
3	IGHV4-4	0.72	0.019	Immunoglobulin heavy chain V-II region	13.016
4	IGKV4-1	0.774	0.028	Immunoglobulin kappa variable 4-1	13.38
5	IGHV3-15	0.789	0.018	Immunoglobulin heavy variable 3-15	12.926
6	IGHV3-7	0.799	0.017	Immunoglobulin heavy variable 3-7	12.943
7	IGHV1-2	0.833	0.011	Immunoglobulin heavy variable 1-2	13.085
8	BLVRB	0.802	0.028	Biliverdin reductase B	22.119
9	PDIA3	0.809	0.014	Protein disulfide-isomerase A3	56.782
10	TPM1	1.204	0.033	Tropomyosin alpha-1 chain	25.47
11	TPM3	1.336	0.022	Tropomyosin alpha-3 chain	33.222
12	GBA	1.207	0.002	Glucosylceramidase	50.263
13	DSG2	1.214	0.010	Desmoglein-2	122.29
14	SRC	1.296	0.013	Proto-oncogene tyrosine-protein kinase Src	59.834
15	CHGA	1.352	0.000	Chromogranin-A	50.688
16	ITGB3	1.376	0.027	Integrin beta 3	86.869
17	TAGLN2	1.441	0.026	Transgelin-2	21.086

^a^Differentially expressed proteins and were defined as having fold changes (Longevous/Normal ratio) > 1.2 (> 1.20 increased or < 0.83 decreased). A two tail Student’s T-test was performed and a p-value < 0.05 was considered to be statistically significant

^b^MW, molecular weight of the differentially expressed proteins

For an overview of the aforementioned DEPs, ontological functions were explored using UniProt-GOA. The ontology of biological processes indicated that these proteins were involved in 16 biological processes including single-organism process, response to stimulus, signaling, multicellular organismal process, multi-organism process, cellular process, cellular component organization or biogenesis, biological regulation, developmental process, reproduction, immune system process, biological adhesion, locomotion, localization, metabolic process and cell killing (Fig. 1c). For cellular components, Fig. 1d illustrated that the proteins were main membrane, cell, extracellular region, organelle and cell junction components. The ontology of molecular functions suggested that the represented function of the DEPs were binding and catalytic activity (Fig. 1e).

### Plasma autoantibody identification by human proteome microarray

**Fig. 2:**
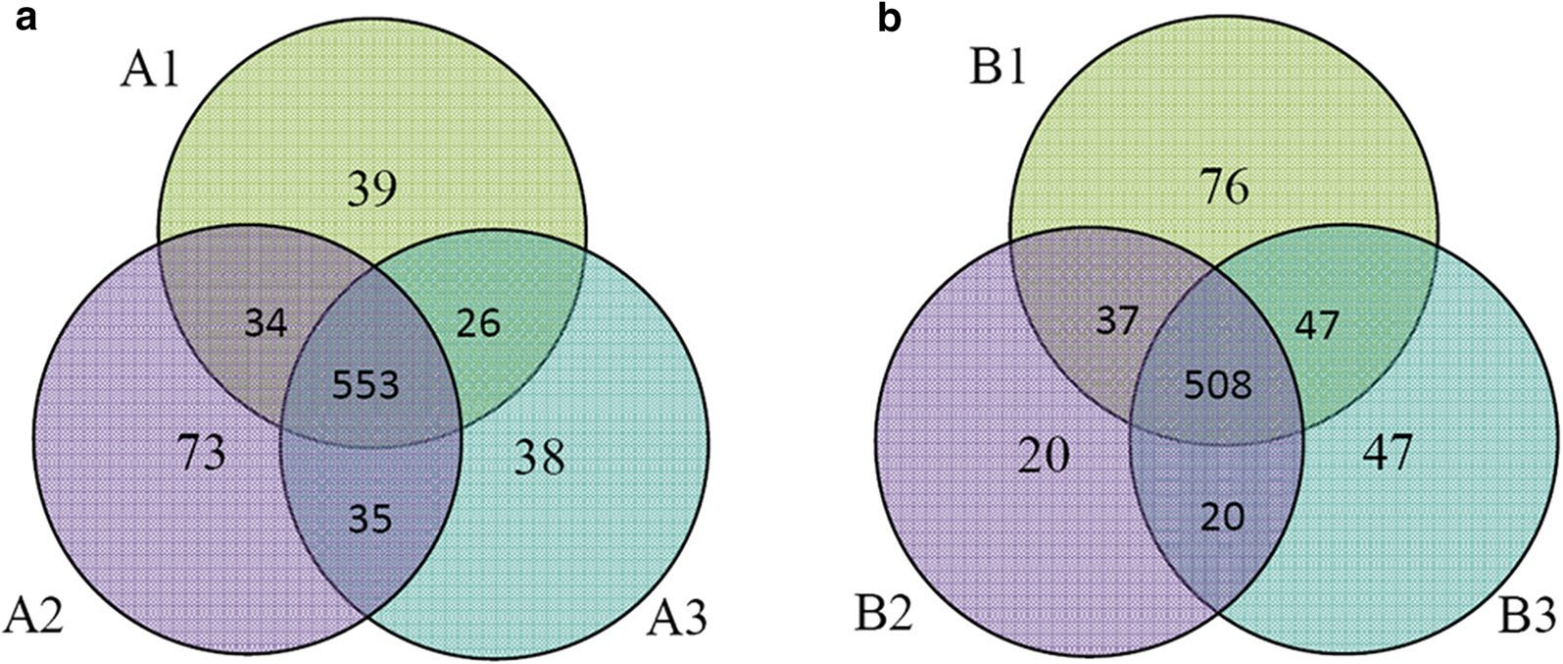
Venn diagram of the number of the immune-reactive autoantigens in the Longevous and Normal groups identified by human proteome microarray. **a** Longevous group, A1, A2 and A3, 3 replicates of offsprings of Bama longevous families; **b** Normal group. B1, B2 and B3, 3 replicates of offsprings of Bama non-longevous families

The HuProt arrays were employed to profile the immune-reactive autoantigens of the two groups of people from Bama longevity area. The 6 samples were individually incubated on the HuProt arrays, followed by incubation with a fluorescently labeled secondary anti-human IgG antibody to identify immune-reactive autoantigens. Using a relatively stringent cutoff value, human protein positively reacted to each sample were identified as the autoantigens that reacted with the autoantibodies in the 6 pooled plasma samples of the two groups. Finally, 652, 695 and 652 proteins on the human proteome microarray were recognized by the IgG in the 3 samples of the longevous group respectively. Among these proteins, 553 were recognized by all the 3 samples (Fig. 2a and Additional file 2). For the normal group, 668, 585 and 622 autoantigens were identified that directly reacted with the 3 samples respectively, and 508 proteins were recognized by all the samples of the normal group (Fig. 2b and Additional file 2).

**Fig. 3:**
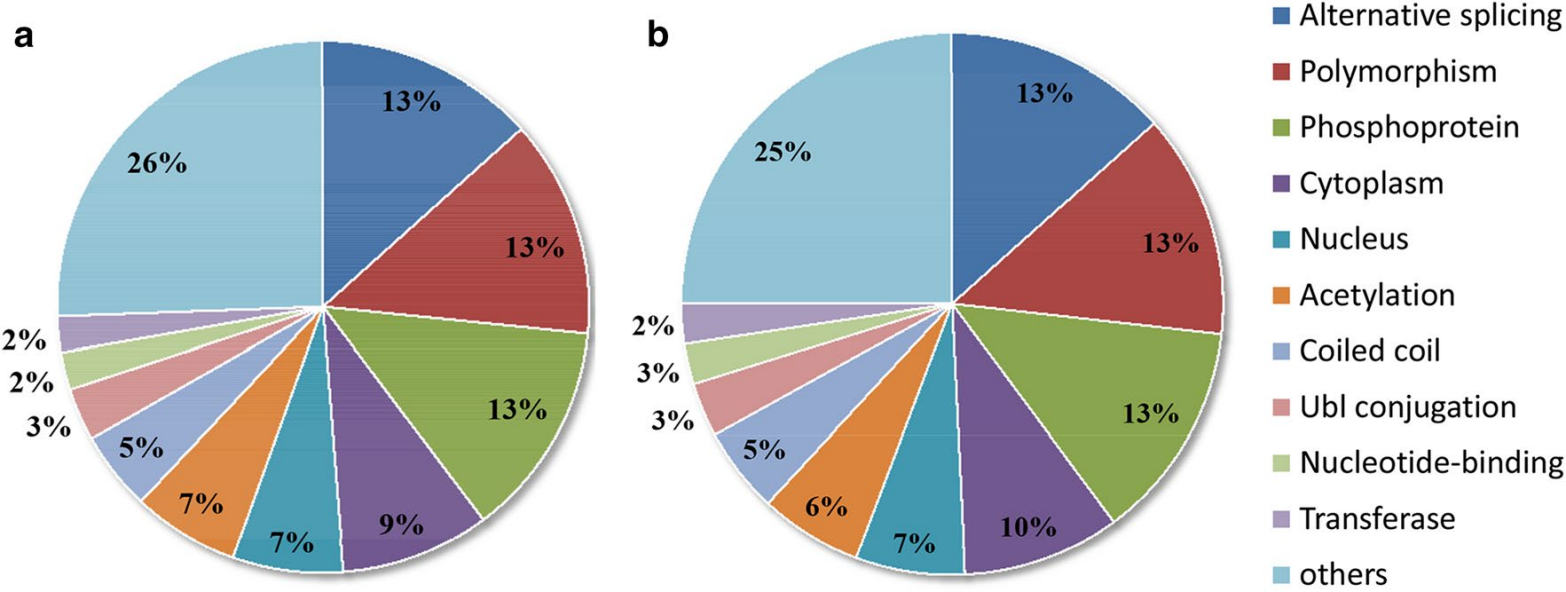
Functional category analyzation of the indentified proteins (autoantigens) recognized by plasma autoantibodies, using human proteome microarrays. **a** 553 autoantigens recognized by all the 3 samples of longevous group; **b** 508 autoantigens recognized by all the 3 samples of normal group

Functional category of the aforementioned 553 (in the longevous group) and 508 (in the normal group) autoantigens were then analyzed. The top 10 of the count number of terms were displayed by pie chat (Fig. 3). The result indicated that the functional category of the antigens recognized by the two groups are almost the same: more than 200 identified antigens were involved in polymorphism (284/258, 13%), alternative splicing (281/258, 13%) and phosphoprotein (278/251, 13%). And other significant function groups included response to cytoplasm (9–10%), nucleus (7%), acetylation (6–7%), coiled coil (5%), Ubl conjugation (3%), nucleotide-binding (2–3%) and transferase (2%), etc.

**Fig. 4:**
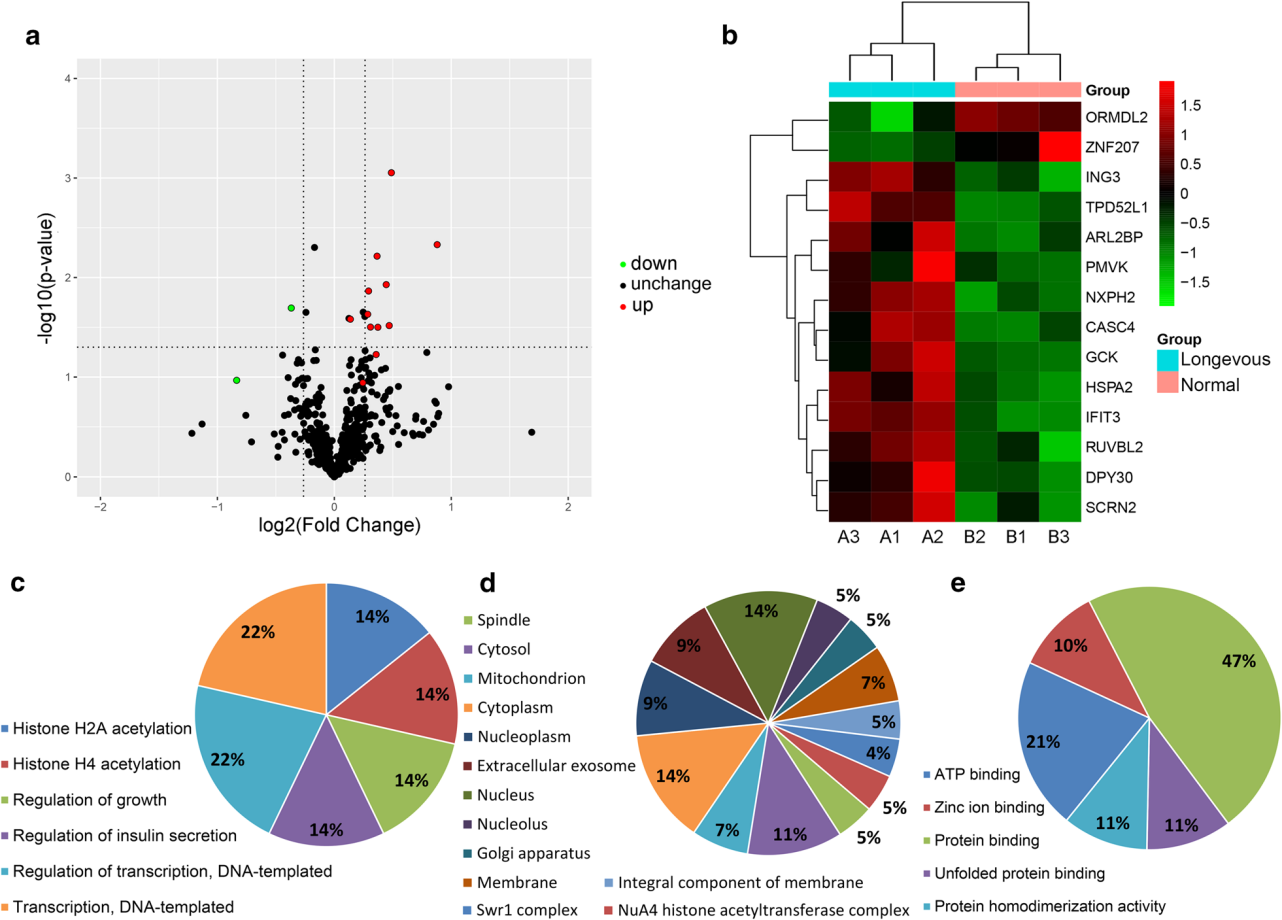
Identified proteins differentially recognized by plasma autoantibodies between the longevous and normal groups. Human proteome microarrays were used to study the autoantibody characteristics in longevity families. A protein recognized in all three samples in one group and not recognized by any sample in another group was defined as a specifically autoantigen that represented a specifically expressed autoantibody. And an antibody with its antigen–antibody reactive signal intensity of featuring a fold change of > 1.2 (> 1.20 increased or < 0.83 decreased) between the two groups was regarded as a differentially expressed autoantibody. A two tail Student’s T-test was performed and a p-value < 0.05 was considered to be statistically significant. **a** The volcano plot shows the up- (red) or down-regulated (green) autoantibodies between the two groups. **b** Hierarchical clustering of specifically and differentially expressed autoantibodies. The color scale bar locates in the bottom, and green and red indicate decreased and increased levels of the identified autoantibodies, respectively. A1, A2 and A3, 3 replicates of the longevous group; B1, B2 and B3, 3 replicates of the normal group. **c** Biological processes, **d** cellular components and **e** molecular functions of GO annotation based function classification of the specifically and differentially expressed autoantibodies

For the determination of the specifically and differentially expressed autoantibodies in the offsprings from longevous and non-longevous families from Bama, all antigens recognized by IgG in plasma samples of the longevous group were compared with that of the normal group. A protein recognized in all three samples in one group and not recognized by any sample in another group was defined as a specific autoantigen. And an antibody with its antigen–antibody reactive signal intensity of featuring a fold change of > 1.2 or < 0.83 (p < 0.05) between the two groups was regarded as a differentially expressed autoantibody. The differentially recognized autoantigens were showed by a volcano plot and a hierarchical clustering heat map (Fig. 4a, b), and described in Table 3. 14 autoantigens were identified, including 6 autoantigens specifically reacted with the sample of the longevous group (CASC4, DPY30, PMVK, HSPA2, IFIT3, SCRN2), 1 specifically reacted with the sample of the normal group (ZNF207), and 7 were differentially recognized by the two groups (ARL2BP, GCK, ING3, NXPH2, RUVBL2, TPD52L1, ORMDL2). Furthermore, 12 of the 14 identified antigens were specifically or more intensively reacted with antibodies in the longevous group.

**Table 3:** Identified proteins (autoantigens) differentially recognized by plasma autoantibodies of the longevous and normal groups, using human proteome microarrays

No.	Name	Ratio^a^	p-value^a^	Protein description	Subcellular location
1^b^	CASC4	1.10	0.026	Cancer susceptibility candidate 4	Cytoplasm
2^b^	DPY30	1.28	0.029	Histone methyltransferase complex regulatory subunit	Nucleus
3^b^	PMVK	1.18	0.044	Phosphomevalonate kinase	Cytoplasm
4^b^	HSPA2	1.23	0.014	Heat shock protein family A (Hsp70) member 2	Nucleus
5^b^	IFIT3	1.40	0.001	Interferon induced protein with tetratricopeptide repeats 3	Cytoplasm
6^b^	SCRN2	1.29	0.032	Secernin 2	Cytoplasm
7^c^	ZNF207	0.56	0.048	Zinc finger protein 207	Nucleus
8^d^	ARL2BP	1.38	0.030	ADP ribosylation factor like GTPase 2 binding protein	Extracellular
9^d^	GCK	1.24	0.031	Glucokinase	Cytoplasm
10^d^	ING3	1.36	0.012	Inhibitor of growth family member 3	Nucleus
11^d^	NXPH2	1.29	0.006	Neurexophilin 2	Cytoplasm
12^d^	RUVBL2	1.22	0.023	RuvB like AAA ATPase 2	Cytoplasm
13^d^	TPD52L1	1.84	0.005	Tumor protein D52-like 1	Extracellular
14^d^	ORMDL2	0.77	0.020	ORMDL sphingolipid biosynthesis regulator 2	Cytoplasm

^a^Human proteome microarrays were used to study the autoantibody characteristics in longevity families. A protein recognized in all three samples in one group and not recognized by any sample in another group was defined as a specifically autoantigen that represented a specifically expressed autoantibody. And an antibody with its antigen–antibody reactive signal intensity of featuring a fold change (Longevous/Normal ratio) of > 1.2 (> 1.20 increased or < 0.83 decreased) between the two groups was regarded as a differentially expressed autoantibody. A two tail Student’s T-test was performed and a p-value < 0.05 was considered to be statistically significant

^b^Autoantigens specifically reacted with IgG in the longevous group (autoantigens reacted with all 3 samples in the longevous group and not reacted with any samples in the normal group)

^c^Autoantigens specifically reacted with IgG in the normal group (autoantigens reacted with all 3 samples in the normal group and not reacted with any samples in the longevous group)

^d^Differential autoantibodies (presented as autoantigens) that had a fold change between the two groups of > 1.2 or < 0.83 and a p value of < 0.05

GO annotation of these specific and differential autoantigens were then explored. The ontology of biological process indicated that these proteins were main involved in 6 processes (Fig. 4c): ING3 and RUVBL2 were participated in the processes of histone H2A acetylation, histone H4 acetylation and regulation of growth, GCK and ARL2BP were related to the regulation of insulin secretion, ING3, RUVBL2, ZNF207 and RUVBL2 were involved in transcription or regulation of transcription. The cellular components ontology showed the identified antigens were located in cytoplasm, nucleus, cytosol, nucleoplasm, extracellular exosome, mitochondrion, nucleolus, membrane, integral component of membrane, Golgi apparatus, etc. (Fig. 4d). The main molecular functions of these antigens were binding, including identical protein binding, ATP binding, zinc ion binding, unfolded protein binding, etc. In addition, DPY30 and TPD52L1 had protein homodimerization activity (Fig. 4e).

## Discussion

Human longevity was considered to be the result of numerous interacting factors including genetic, environmental and behavioural components [31–33]. Several studies suggested that about 20–50% of the variation in human lifespan is accounted for by genetic factors [2, 34, 35]. A moderate clustering of extreme longevity within families was also reported [36, 37]. In the last 30 years, genome and transcriptome studies were employed and many candidate genes have been investigated for putative associations with human survival or longevity. The main categories of the genes are involved in metabolic and immune systems [5]. But little is known about the proteomic profiles involved in longevity. In addition to the whole genome association studies, proteomic analysis can be taken to investigate the molecular mechanism of longevity more accurately, as proteomic profiles do not accurately predicted by transcriptome profiles [6, 38].

For a further investigation of the potential mechanisms of longevity at the protein level, people from a well-known longevous area (Bama, China) were recruited to participate in this study. Offsprings of longevous families were recruited as case studies and ABO-, age- and gender-matched subjects from non-longevous families were selected as controls. TMT-based proteomic technique and human proteome microarrays were used to study the protein and autoantibody characteristics in longevity families. Differentially expressed proteins and autoantibodies were defined as having fold changes > 1.2 and < 0.83, respectively. In addition, a two tail Student’s T-test was performed and a p value < 0.05 with no strategy of correction for multiple testing was considered to be statistically significant based on the previous proteomic and microarray studies [30, 39–42].

### Differentially expressed proteins revealed by quantitative proteomic analysis

By using TMT-based quantitative proteomic analysis, a set of low-abundance plasma proteins were quantified and 12 proteins were found that differentially expressed between the longevous and normal groups. Identified differences indicate involvement of several physiological pathways, such as immunity, metabolism, cell adhesion and signaling. Lipoprotein(a) (LPA) is known as a risk factor for atherosclerotic diseases such as coronary heart disease and stroke [43, 44]. Genetic and epidemiologic studies also showed that individuals without LPA or with very low LPA levels seem to be healthy [45], genes related to cardiovascular health may be implicated in exceptional longevity [5]. Interestingly, LPA was found to be down-regulated most in offsprings from longevous families in our results. Two components involved in immunity were found to be down-regulated in the longevous group : defensin 3 (DEFA3) is expressed primarily in neutrophils as well as in NK cells and certain T-lymphocyte subsets, plays a role in phagocyte-mediated host defense and is considered as a part of the innate immune response [46]. Protein disulfide-isomerase (PDIA3) is part of the major histocompatibility complex (MHC) class I peptide loading complex, which is essential for formation of the final antigen conformation and export from the endoplasmic reticulum to the cell surface [47]. PDIA3 also involved in cytokine-dependent signal transduction, overexpression of it is reported linked to renal fibrosis [48, 49]. Biliverdin reductase B (BLVRB), a NADPH-dependent oxidoreductase, was also low expressed in the offsprings of longevous families. BLVRB is considered to be a critical regulator of cellular redox, as it can physiologically regenerates bilirubin in a catalytic cycle [50].

Several proteins were found to be up-regulated in offsprings from longevous families. Integrin beta 3 (ITGB3, CD61) are known to participate in cell adhesion as well as cell-surface-mediated signaling [51, 52], chromogranin A (CHGA) is widely expressed by neuroendocrine cells, induces and promotes the generation of secretory granules [53]. Proto-oncogene tyrosine-protein kinase Src may play a role in the regulation of embryonic development and cell growth, an elevated level of activity of this protein is suggested to be linked to cancer progression by promoting other signals [54]. Desmoglein-2 (DSG2) is localized to desmosome structures at regions of cell–cell contact and functions to structurally adhere adjacent cells together. Tropomyosin (TMP) is involved in the cytoskeleton of non-muscle cells.

Several proteomic studies have been reported to reveal the age-related protein changes in plasma, cerebral and serum [55–58]. One study that was conducted in plasma of women enrolled in the TwinsUK study. SOMAscan protein data of 206 female twins were analyzed. Thirteen age‐associated proteins were discovered. Some of them were previously been described to related to cardiovascular disease, apoptosis and aging-associated neuroinflammation [55]. Another SOMAscan proteomic study identified 217 age‐associated plasma proteins,which were main involved in blood coagulation, chemokine and inflammatory pathways, axon guidance, peptidase activity, and apoptosis [56]. 12 of the 13 age-associated proteins reported in TwinsUK study were confirmed to be associated with age in the latter study. In our study, none of the differentially expressed plasma proteins between the longevity and normal group were found to be consistent with the results of the two studies. It is difficult to directly compare the results from the two studies and our work. Because in this study two groups of age-matched individuals were enrolled. And the subjects were grouped based on family longevity, not age.

### Plasma autoantibody identification by human proteome microarray

Human plasma contains a large number of autoantibodies that recognize self proteins in healthy individuals, and the autoreactive repertoires are predominantly selected during fetal life [25]. Several functions have been proposed for autoantibody, such as defense against infection, clearance of aging cells, anti-tumoral surveillance and anti-inflammatory activity [25, 26]. In the second part of our study, by using human proteome microarray, more than 500 proteins were found to be recognized by plasma autoantibodies. 14 proteins ware differentially reacted with the autoantibodies in the longevous and normal groups. In addition, we found 12 proteins have higher affinity with antibodies or specifically react with antibodies in plasma of longevous group. Among the 12 proteins, 5 are reported to be related to cancer: CASC4 is a cancer susceptibility candidate, reduction of the expression of CASC4 has been shown to suppress the proliferation of gastric cancer [59]; tumor protein D52-like 1 (TPD52L1) is a cell cycle-regulated protein and maximally expressed at the G2-M transition in breast cancer cells [60]; secernin 2 (SCRN2) is found to be a tumor-associated antigen in gastric cancer [61]; inhibitor of growth family member 3 (ING3) is a member of the ING tumor suppressor family, overexpression of this gene has been shown to inhibit cell growth and induce apoptosis [62]; RuvB like AAA ATPase 2 (RUVBL2) plays roles in transcriptional regulation and DNA repair, it also concerns cancer-related processes via interaction with oncogenic transcription factors [63, 64]. Other autoantigens were also involved in some important biological processes: glucokinase (GCK) and phosphomevalonate kinase (PMVK) are metabolic enzymes that play important role in the carbohydrate metabolism and mevalonate pathway respectively. Histone methyltransferase complex regulatory subunit (DPY30) is located on the nucleus and acted as a transcription regulator [65]. Heat shock protein (HSPA2) protects cells from oxidative stress and inhibits apoptosis [66]. And interferon induced protein (IFIT3) takes part in cell signaling associated with the immune system [67]. In addition, only two proteins, a Zinc finger protein (ZNF207) and an endoplasmic reticulum-localized transmembrane protein (ORMDL2), were found specifically and more intensively reacted with IgG in the normal group.

## Conclusions

By using TMT-based proteomic technique and human proteome microarray, the differences of proteomics and autoantibody profiles between offsprings from longevity and non-longevity families in Chinese Bama longevous area were investgated. As a result, 12 proteins and 14 autoantibodies were discovered differentially expressed between the longevous and normal groups. Most of the differential proteins and targeted autoantigens were reported playing critical roles in some physiological and pathological processes. These findings will be contributed to a better understanding of the proteomic characteristics of people from Bama longevous area and a revelation of the mystery of longevity. Further studies are needed to study the effect and molecule mechanism of the differential expressed proteins and autoantibodies on longevity.

## Additional files

**Additional file 1. Table S1.** Low abundance proteins in the the plasma of the longevous and normal groups, identified by TMT-based quantitative proteomic analysis.

**Additional file 2. Table S2**. Proteins (autoantigens) recognized by plasma autoantibodies of the longevous and normal groups, identified by human proteome microarray.

## Data Availability

The datasets supporting the conclusions of this article are included within the article and its additional files.

## Abbreviations

DEP: differentially expressed protein
FDR: false discovery rate
GO: gene ontology
GST: glutathione S-transferase
IgG: immunoglobulin G
MHC: major histocompatibility complex
TMT: tandem mass tag
